# Chaining of hard disks in nematic needles: particle-based simulation of colloidal interactions in liquid crystals

**DOI:** 10.1038/s41598-020-69544-4

**Published:** 2020-07-29

**Authors:** David Müller, Tobias Alexander Kampmann, Jan Kierfeld

**Affiliations:** Physics Department, TU Dortmund University, 44227 Dortmund, Germany

**Keywords:** Soft materials, Colloids, Liquid crystals, Self-assembly, Materials science, Physics, Chemical physics, Statistical physics, thermodynamics and nonlinear dynamics, Computational science

## Abstract

Colloidal particles suspended in liquid crystals can exhibit various effective anisotropic interactions that can be tuned and utilized in self-assembly processes. We simulate a two-dimensional system of hard disks suspended in a solution of dense hard needles as a model system for colloids suspended in a nematic lyotropic liquid crystal. The novel event-chain Monte Carlo technique enables us to directly measure colloidal interactions in a microscopic simulation with explicit liquid crystal particles in the dense nematic phase. We find a directional short-range attraction for disks along the director, which triggers chaining parallel to the director and seemingly contradicts the standard liquid crystal field theory result of a quadrupolar attraction with a preferred ${45^{\circ }}$ angle. Our results can be explained by a short-range density-dependent depletion interaction, which has been neglected so far. Directionality and strength of the depletion interaction are caused by the weak planar anchoring of hard rods. The depletion attraction robustly dominates over the quadrupolar elastic attraction if disks come close. Self-assembly of many disks proceeds via intermediate chaining, which demonstrates that in lyotropic liquid crystal colloids depletion interactions play an important role in structure formation processes.

## Introduction

Colloidal suspensions of nanometer to micrometer-sized particles in a host fluid can form liquid and crystalline phases, but also liquid crystalline mesophases if colloidal particles are, for example, rod-shaped^1, 2^. Phase behavior and stability of a colloidal system can often be explained based on effective interactions between colloidal particles which arise from integrating out microscopic degrees of freedom of the host fluid. The resulting effective interactions govern colloidal stability, coagulation, flocculation, and structures that eventually self-assemble.

Colloidal mixtures containing different colloidal particles, often of different size, feature additional effective interactions if degrees of freedom of one species are integrated out^3^. The most prominent example of additional effective interactions in mixtures are depletion interactions, as they arise, for example, in a mixture of large and small hard spheres^4, 5^: a short-range attractive depletion interaction between large hard spheres emerges because the excluded volume for the small spheres decreases (and, thus, their available phase space increases) if large spheres approach closer than one diameter of a small sphere.

Effective interactions become even more interesting for colloids suspended in anisotropic fluids such as liquid crystals (LCs), which can also be seen as colloidal mixtures of larger colloidal particles suspended in a liquid of small rod-like particles, in particular for lyotropic LCs^6^. Such LC colloids exhibit anisotropic effective interactions between colloidal particles if the LC is in an ordered, e.g., nematic phase^7^. The first studies of LC colloids have been performed on latex spheres suspended in a lyotropic nematic LC (a micellar nematic phase of discoid type)^8, 9^. The nematic LC phase forms typical defect-structures around a spherical inclusion depending on the anchoring conditions, “Saturn-ring” disclination rings or a satellite hedgehog for normal anchoring and boojums for planar anchoring^10–12^. These defect-structures also induce long-range colloidal interactions between spherical inclusions. These have been first explored systematically by Ramaswamy *et al.*^13^, Ruhwandl and Terentjev^14^ and by Poulin *et al.* who also observed a chaining of water droplets inside a LC^15^. The nature of these elastic LC-mediated interactions strongly depends on the details of the interaction between colloidal particle and LC host, i.e., how the LC molecules or rods are anchored on the colloid (normal, conic or planar, weak or strong anchoring), and can be of dipolar, quadrupolar, or even more complicated nature^16–18^. Such systems are promising candidates to realize a controlled self-assembled structure formation by anisotropic interactions^19–24^. The elastic interactions in combination with different shapes and sizes of the colloidal particles can be used to create a multitude of different colloidal interactions^25–27^. LC colloidal assemblies can be engineered, for example, by tuning the surface anchoring or engineering nematic defect structures^24,28, 29^.

Here, we consider a colloidal mixture of hard disks and hard needles, which serves as a simple two-dimensional model system of colloidal spheres suspended in a lyotropic LC. We note that systems of had rods and hard disks in three dimensions are qualitatively different as both disks and rods form LC phases in three dimensions^30,31^. We are interested in the situation, where the hard needles are sufficiently dense to form a nematic LC phase such that they can mediate elastic interactions between the hard disks. This system is interesting from a theoretical point of view as it can be interpreted, on the one hand, as a colloidal mixture, where we expect depletion interactions between two hard disks embedded into a fluid of shorter needles. We also expect, on the other hand, to find long-range elastic interactions mediated by the nematic hard needle LC, at least for needles shorter than the disks, such that a coarse-grained continuum description is appropriate. This is the situation we want to address in this paper. The interplay and competition of (at least) two types of effective interactions—short-range depletion and long-range elastic—has to be unraveled and will have interesting consequences for the total effective interaction between the disks.

Depletion interactions are well documented for the dilute isotropic phase of needles^32–37^ but much less is known for a nematic host with the notable exceptions of Refs.^38, 39^, where the limit of small spheres in long needles has been considered. In the experimental study in Ref.^39^ a chaining of small spherical particles in a host of fd virus rods parallel to the director has been found, which was attributed to depletion attraction.

On the other hand, the theory of colloidal LCs should apply with long-range elastic interactions mediated by director field distortions in the nematic hard needles. Depending on anchoring conditions and dimensionality of the system dipolar interactions (falling off as $r^{-3}$ with the sphere separation in three dimensions)^40–42^ or quadrupolar interactions (falling off as $r^{-5}$) can occur^13, 14, 43–45^. Because hard needles tend to align tangentially at a hard wall, we expect a quadrupolar elastic interaction, which is characteristic for planar anchoring at the colloidal disk^13, 14, 44–46^ but also generic in two dimensions^43^. Both for dipolar and quadrupolar interactions the elastic interaction is attractive and chaining of colloidal spheres has been experimentally observed^20, 47, 48^. Whereas dipolar interactions prefer chaining of spheres parallel to the director axis in three dimensions^41, 42^, quadrupolar interactions prefer an angle of approximately ${30^{\circ }}$ with respect to the director axis in three dimensions^44, 46^ and a ${45^{\circ }}$ angle in two dimensions^45^. For our two-dimensional system of hard disks and hard needles we thus expect the elastic interaction to favor a ${45^{\circ }}$ angle if disks form chains.

Colloidal mixtures are also computationally challenging systems. Effective interactions are essential to characterize stability and potential self-assembly into crystalline phases but hard to access in a microscopic particle-based simulation. The process of integrating out microscopic degrees of freedom corresponds to the numerical evaluation of a potential of mean force between the colloidal species of interest. In order to measure the potential of mean force all degrees of freedom including, for example, relatively slow large spheres in a bath of small particles must be properly equilibrated.

The colloidal mixture of hard disks suspended in a nematic host of hard needles is particularly challenging as the hard needle system must be fairly dense to establish a nematic phase. While particle-based simulations exist for dilute rods in the isotropic phase^49, 50^, the regime of a nematic host is fairly unexplored up to now and simulations resorted to coarse-graining approaches^46^. So far, only single inclusions^51^ or confining geometries^52^ have been investigated by particle-based simulations. In order to calculate the effective interactions between hard disks and their self-assembly we apply a novel Monte Carlo (MC) technique, the rejection-free event-chain sampling technique. The event-chain algorithm has been originally proposed for pure hard sphere systems^53^, and recently generalized to dense hard needle systems^54^, and is used here to efficiently equilibrate the system, which allows us to directly obtain the potential of mean force and, thus, the effective interaction between two hard spheres in a nematic hard needle host.

The simulation will reveal a surprising and robust tendency for chaining of disks along the director axis which seems to contradict the chaining in a ${45^{\circ }}$ angle with respect to the director axis as predicted by quadrupolar elastic interactions in two dimensions^45^. This turns out to be a result of a dominant short-range depletion interaction, and the analysis and explanation of this phenomenon are the main topic of the present paper.

**Figure 1:**
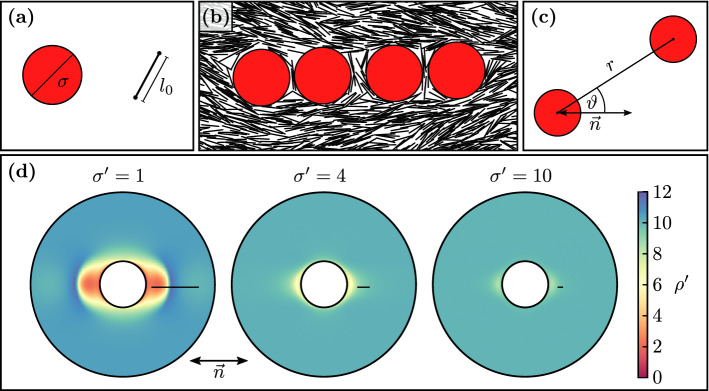
(**a**) Simulated particles: hard disks with diameter $\sigma$ and hard needles with characteristic length $l_0$. (**b**) Simulation snapshot of disks ($\sigma /l_0=3$) forming chains along the director $\vec {n}$ (approximately in horizontal direction). On the outer disks and between disks surface depletion zones with low needle density form. (**c**) Disk distance *r* and disks angle $\vartheta$ towards the director $\vartheta$ are used to describe the potential of mean force as well as other interactions. (**d**) Needle center of mass density distribution $\rho '(\vec {r})$ around a disk, relative to the director for different relative disk sizes $\sigma '=\sigma /l_0$ (scale bars are one needle length $l_0$). For small disks, there are distinct depletion zones in front of and behind a disk. Depletion zone extensions are $O(l_0)$. Overlap of these depletion zones gives rise to the density-dependent depletion interaction. (Figure created using gnuplot 5.2 (http://www.gnuplot.info/), matplotlib 3.2.1 (https://matplotlib.org/), python 3.8.2 (https://www.python.org/), inkscape 0.92.5 (https://inkscape.org/)).

## Results

We use hard needles as a model system for a lyotropic LC. Hard needles can be viewed as two endpoints connected by an infinitely thin, hard line, see Fig. 1a, and we sample in the MC simulation by moving the two endpoints. The needle length $l_0$ is used as unit length for non-dimensionalization in the following (primed quantities are dimensionless). In two dimensions needles can order at sufficiently high area densities $\rho _\text {n}'\equiv \rho _\text {n} l_0^2 > 6$ in a Kosterlitz-Thouless transition into a quasi-ordered nematic phase^54–56^. In the following, we focus on a needle system with $\rho _\text {n}'= 10$ well within this nematic regime. We suspend hard disks of diameter $\sigma$ (${\sigma '\equiv \sigma /l_0}$) as colloidal particles into the nematic hard needle system, see Fig. 1b. The interaction between all particles, i.e., disk-disk, disk-needle and needle-needle, is given by hard core potentials, which prohibit overlaps. For fast sampling, we employ the rejection-free event-chain MC algorithm. More details on the simulation are provided in the Methods section. The resulting needle-mediated effective interactions between disks in this lyotropic system is the focus of our investigation.

### Chaining and depletion

In the MC simulation, we observe a chaining of the disks along the director axis of the nematic needle phase, see Fig. 1b. This chaining indicates a strongly anisotropic directional attraction caused by the hard needles along the director axis. The chaining axis coincides with two elongated zones depleted of needles in the direction of the director on the surface of the disk, as revealed by the needle density shown in Fig. 1d. Depletion zones have an extension of order $l_0$. Therefore, they are deep relative to the disk diameter and very distinct for small disks (${\sigma '=1}$). For larger disks, the depletion zones smear out and their depth diminishes as compared to the disks size.

**Figure 2:**
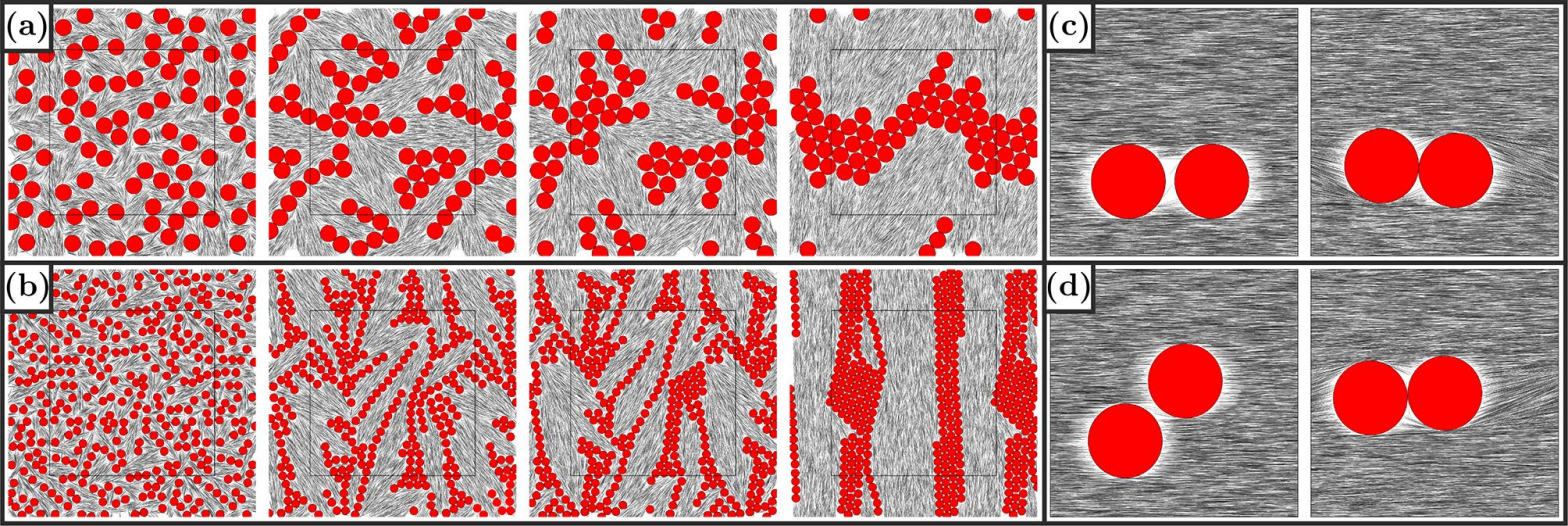
(**a**) and (**b**) Evolution of a multi-colloid system, containing 40 disks with diameter $\sigma = 2$ in (**a**) 160 disks with diameter $\sigma = 1$ in (**b**). In both systems the same area is covered by disks, the needle density is $\rho '_\text {n} = 20$. To generate the initial configuration we place the disks overlap-free and add needles where they fit until the desired density is achieved. The series of snapshots shows the self-assembly of the disks first in chains and then into hexagonal clusters. We show partly periodic images, extending the original system indicated by a black square. (**c**) and (**d**) Also in high density systems ($\rho '_\text {n}=100$) disks align with the director. (**c**) and (**d**) have different initial configurations, shown in the left column with disks at 0 and 45 angles. Right column shows equilibrated state. (Figure created using gnuplot 5.2 (http://www.gnuplot.info/), inkscape 0.92.5 (https://inkscape.org/)).

Chaining along the director axis also governs self-assembly in simulations containing many disks shown in Fig. 2a,b. Here we observe self-assembly of hexagonal disk crystals via intermediate chain formation and subsequent aggregation of parallel chains. The chaining along the director axis is very robust and occurs up to high needle densities $\rho '(n)=100$, see Fig. 2c,d, where the nematic LC becomes very stiff.

### Effective disk-disk interaction

**Figure 3:**
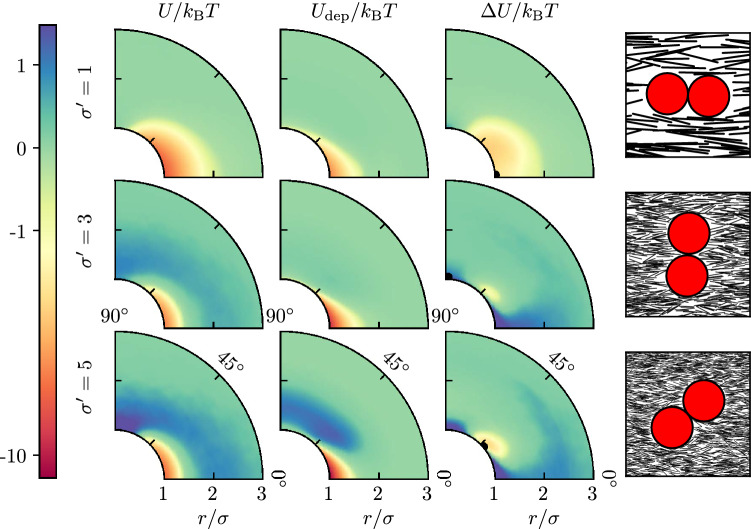
Contour plots of the measured interaction *U*, the calculated density-dependent depletion interaction $U_\text {dep}$ and the difference of the two $\Delta U = U - U_\text {dep}$, showing the quadrupolar behavior. Columns represent the different interactions and rows describe different disk diameters $\sigma ' = 1$, 3 and 5. The measurement was performed in a square system $L \times L$ with system size $L=10 \sigma$ and needle density $\rho _\text {n}'=10$. On the right side are exemplary snapshots of configurations, marked with the black dot in the contour plot next to it. (Figure created using matplotlib 3.2.1 (https://matplotlib.org/), python 3.8.2 (https://www.python.org/), inkscape 0.92.5 (https://inkscape.org/)).

To quantify the effective interaction between two hard disks suspended in nematic hard needles, we use the potential of mean force ${U(r, \vartheta )\approx - k_\text {B} T \ln g(r, \vartheta )}$. Here, *r* is the distance between the disks and $\vartheta$ the angle between the connecting line and the director $\vec {n}$, see Fig. 1c; $g(r, \vartheta )$ is the pair distribution function and $k_\text {B} T$ the thermal energy. The event-chain MC simulation technique is ideally suited for dense mixed colloidal systems^54, 57^ and allows us to obtain the positional and angular dependence of the potential of mean force as shown in Fig. 3.

For small angles $\vartheta$ the interaction is attractive and has its minimum at the disk surface at an angle of ${\vartheta =0^{\circ }}$. This explains the observed parallel chaining of disks. For larger disks, the interaction also develops a repulsive part around ${\vartheta =90^{\circ }}$. The range of the interaction is decreasing relative to the disk size and of order of $l_0$.

The effective interaction can be described as the sum of two interactions, which are the depletion interaction of short range $l_0$ resulting from overlapping depletion zones around a hard disk, and a power-law quadrupolar elastic interaction resulting from the elastic energy of nematic hard needles. Because hard needles tend to align tangentially at a hard wall, we expect a quadrupolar elastic interaction falling off as $r^{-4}$ and with a $\cos (4\vartheta )$ angular characteristic^43, 45, 58^. Therefore, the elastic interaction is maximally attractive for ${\vartheta =45^{\circ }}$ and repulsive both for ${\vartheta =90^{\circ }}$ and ${\vartheta =0^{\circ }}$. The repulsive interaction at ${\vartheta =90^{\circ }}$ can thus be explained by dominating quadrupolar elastic interactions, which becomes more relevant for larger disks. The interaction minimum at ${\vartheta =0^{\circ }}$, however, comes as a surprise in view of the quadrupolar interaction of standard LC field theory. This minimum can only be explained by the dominance of the attractive depletion interaction induced by the nematic hard needles, which must be directed along the depletion zones parallel to the director.

### Density-dependent depletion interaction

In order to calculate the depletion interaction caused by the complex anisotropic and smeared shapes of the depletion zones in Fig. 1d, we use a density-dependent description of depletion interactions and find

1 $$\begin{aligned} U_\text {dep}(\vec {r})&= - k_\text {B} T \rho _\text {n} \int \left( 1 - \frac{\rho (\vec {r}')}{\rho _\text {n}} \right) \left( 1 - \frac{\rho (\vec {r}'-\vec {r})}{\rho _\text {n}}\right) {\mathrm{d}} \vec {r}', \end{aligned}$$

where $\rho (\vec {r})$ is the local needle density and $\rho _\text {n}$ the average needle density. The depletion interaction is proportional to a generalized overlap area of depletion regions of two disks at distance $\vec {r}$, where the depletion region of a disk at $\vec {r}'=0$ is given by the region with $1 - {\rho (\vec {r}')}/{\rho _\text {n}}\approx 1$, which can correctly capture the complex shaped depletion zones in Fig. 1d. A detailed derivation of Eq. (1) is presented in the Methods section.

The depletion zones are mainly shaped by the elastic interactions in the nematic phase via anchoring conditions at the disk surface. Hard rods exhibit planar anchoring at a hard disk. In the simulation the anchoring is only parallel on average, and fluctuations weaken the anchoring considerably. We only find a weak entropic planar anchoring (quantified below) such that the needle orientation only deviates little from the director orientation at the disk surface. This explains the elongated depletion zones of extension of order $l_0$ (see Fig. 1d). This also results in an overlap area $\sim (\sigma l_0^3)^{1/2}$ from standard geometric arguments^5^, such that $U_\text {dep} \sim k_BT \rho _\text {n}' \sigma '^{1/2}$ is expected. Moreover, this gives rise to a strongly anisotropic depletion attraction, which is maximal parallel to the director ($\vartheta =0^{\circ }$). We can calculate the depletion interaction numerically using measured density profiles from simulations and (1); the result is shown in Fig. 3.

### Revealing the residual elastic interaction

In order to uncover additional effective interactions apart from depletion, we subtract the numerically calculated depletion interaction from the measured total potential of mean force and obtain the residual interaction ${\Delta U = U - U_\text {dep}}$. All three interactions are shown in Fig. 3. This reveals the presence of another interaction, which can actually be identified as the elastic interaction from LC field theory. Hard needles tend to align tangentially at a hard wall, such that we have planar boundary conditions. For two disks suspended in a nematic LC with planar boundary conditions, LC field theory predicts^43, 58^

2 $$\begin{aligned} U_\text {quad}(r, \vartheta )&\approx \frac{3 \pi K \sigma ^4}{4} \frac{\cos (4 \vartheta )}{r^4} \, . \end{aligned}$$

**Figure 4:**
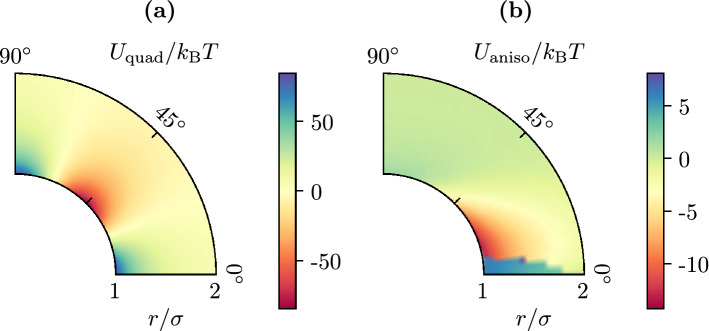
(**a**) Quadrupolar interaction (2) of two disks in a liquid crystal with planar boundary condition on the disk’s surface (with ${K / \rho _\text {n}'^2 k_\text {B}T = 0.358}$, see text). This is a field theoretical result for the far-field behavior, assuming weak distortions of the director and using a one-constant approximation. (**b**) Numerically calculated quadrupolar interaction between two disks in a system of size $L=10\sigma$ in the presence of elastic anisotropy and weak anchoring ($K_1/K_3=0.1$ and $w=0.5$, see (4), and $K_3 / \rho _\text {n}'^2 k_\text {B}T = 0.63$). (Figure created using matplotlib 3.2.1 (https://matplotlib.org/), python 3.8.2 (https://www.python.org/), inkscape 0.92.5 (https://inkscape.org/)).

This result is based on the one-constant approximation, i.e., assuming an isotropic elasticity of the nematic LC with a single elastic constant *K*. This elastic constant can be calculated for hard needles in two dimensions as ${K / \rho _\text {n}'^2 k_\text {B}T = 0.358}$ by adapting a method from Straley^59^. The quadrupolar interaction (2) is shown in Fig. 4a and explained in more detail in the Methods section.

The field-theoretical quadrupole interaction (2) is qualitatively similar to the residual interaction $\Delta U$ from Fig. 3 but exhibits characteristic deviations. The main differences are: (1) a much weaker interaction strength, (2) a distorted quadrupolar symmetry, and (3) for small disks (${\sigma ' = 1}$), a missing repulsive regime around ${\vartheta = 0^{\circ }}$ and ${\vartheta = 180^{\circ }}$. The low interaction strength of the measured interaction (i) is caused by the weak parallel anchoring of hard rods at the disk surface, which we find in the simulation (and which we quantify below). In the theoretical result (2), on the other hand, strict planar boundary conditions, i.e., infinitely strong parallel anchoring is assumed. The distorted quadrupolar symmetry (ii) becomes manifest in differently strong repulsion zones for ${\vartheta =0^{\circ }}$ and ${\vartheta =90^{\circ }}$. This is caused by the elastic anisotropy of the nematic hard needle phase, i.e., different elastic constants ${K_1 \ne K_3}$ for orientation fluctuations perpendicular ($K_1$) or parallel ($K_3$) to the director, which are not captured in the one-constant approximation (${K = K_1 = K_3}$) employed in (2). Adapting the method from Ref.^59^, we find $K_1/K_3 \approx 0.14$ for hard needles in two dimensions (our above value ${K / \rho _\text {n}'^2 k_\text {B}T = 0.358}$ is actually defined as $K=(K_1+ K_3)/2$. From fitting mean nematic orientation field around a disk we confirm a value of $K_1/K_3 \approx 0.1$ below. The weak repulsion for small disks (${\sigma '=1}$) at ${\vartheta =0^{\circ }}$ and ${\vartheta =180^{\circ }}$ (iii) can be explained by the pronounced density dependence of the elastic constant ($K\propto \rho _\text {n}^2$) via a correlation effect with the depletion interaction. Since the depletion zones are of very low density and pronounced in this direction, especially for small disks, they also weaken the elastic interaction in this direction.

This interpretation is supported by a numerical calculation of the elastic interaction between two disks in the presence of elastic anisotropy $K_1/K_3 = 0.1$ and weak parallel anchoring (as quantified below), which is shown in Fig. 4b. Details of the numerical calculation are explained in the Methods section. The numerically calculated elastic interaction only misses the correlation effect (iii) and, indeed, resembles the residual interaction $\Delta U$ from Fig. 3 more closely in its distorted shape.

Both depletion and quadrupolar interaction are attractive and promote chaining of disks, however, at angle $0^{\circ }$ if depletion dominates and around $45^{\circ }$ if the elastic quadrupolar interaction dominates. We see a robust $0^{\circ }$-chaining which points at a dominating depletion interaction. In order to explain this dominance we need to quantify the weak anchoring for the hard needle and disk system, which is central both for directing the depletion interaction in $0^{\circ }$ direction as well as for weakening the residual elastic interaction.

### Weak anchoring strength and elastic anisotropy in quadrupolar interaction

Both anchoring strength and elastic anisotropy can be quantitatively analyzed by investigating the needle orientation field $\Phi (\vec {r})$ around a single disk, which is suspended in a nematic phase with $\Phi =0$ asymptotically at the system boundary ($\Phi (\vec {r})$ is the angle of the local director field $\vec {n}(\vec {r})$ with this asymptotic orientation, i.e., $\vec {n}=(\cos \Phi , \sin \Phi )$; $\Phi$ is defined with respect to the asymptotic director orientation, which determines the *x*-axis). By comparing numerical MC results for hard needles and field theory calculations we can deduce both anchoring strength and elastic anisotropy.

The free energy of a two-dimensional LC with anchoring on a surface is given by the Frank-Oseen elastic energy^60^ and a surface anchoring potential:

3 $$\begin{aligned} F&= \int {\mathrm{d}} A \left[ \frac{K_3}{2} (\partial _x \Phi )^2 +\frac{K_1}{2} (\partial _y \Phi )^2 \right] +\frac{W}{2} \int \sin ^2(\Phi _0 - \Phi ) {\mathrm{d}} l \, . \end{aligned}$$

The first integral is the approximate Frank-Oseen free energy $F^\text {2D}_\text {FO}$ with elastic constants $K_1$ and $K_3$ (see Methods section) and the second integral over the disk’s surface represents the anchoring energy with anchoring strength *W*. The anchoring depends on the boundary condition $\Phi _0$ at the disks surface, which is parallel anchoring for hard needles at a hard disk (i.e., $\Phi _0 = \theta -\pi /2$). The numerical minimization of this free energy is discussed in the Methods section. Introducing dimensionless quantities (marked with a tilde) by measuring the free energy in units of the elastic constant $K_3$ and distances in units of the disk radius $R = \sigma /2$, we find two control parameters, the elastic anisotropy $K_1/K_3$ and the relative anchoring strength $w\equiv W\sigma /2K_3$.

We fit the free energy minimization results to MC simulation data for the mean orientation field $\Phi (\vec {r})$ of the needles (see Methods section). Fitting $\Phi (\vec {r})$ in the range $2r/\sigma =1.5-3$ and doing so for nematic densities $\rho _\text {n}'=10-20$ and disk sizes $\sigma '=1-10$ in systems of size $L=6\sigma$ we obtain the best fit for

4 $$\begin{aligned} \frac{K_1}{K_3}&\approx 0.1 ~~\text{ and } ~~~w\approx 0.45-0.50. \end{aligned}$$

In principle, the relative anchoring strength *w* can depend on $\rho _\text {n}'$ and $\sigma '$. We only find a very weak decrease from $w=0.5$ to 0.45 with density for $\rho _\text {n}'=10-20$. The result for the elastic anisotropy is in good agreement with our above finding $K_1/K_3 \approx 0.14$ using the method from Ref.^59^. Both results (4) are also very similar to results from Ref.^61^ for granular rods in two dimensions obtained in a cavity geometry. This confirms that the hard needle nematic phase has indeed a pronounced elastic anisotropy and only a weak effective anchoring strength at the hard disk surfaces; this anchoring is purely entropic as reflected by $w=\mathrm{const}$ implying $W/\sigma \sim K \sim k_BT \rho _\text {n}'^2$.

### Depletion dominates quadrupolar interaction for chaining along director

In the chaining of disks the elastic quadrupolar interaction competes with the depletion interaction. For weak anchoring the quadrupolar interaction is $\propto w^2$^45^. From Eq. (2) we expect a quadrupolar interaction strength $U_\text {quad}/{k_BT} \sim w^2 \rho _\text {n}'^2$ at the disk surface. This competes with a depletion interaction of strength $U_\text {dep}/k_BT \sim \rho _\text {n}' \sigma '^{1/2}$. For weak anchoring with $w^2\rho _\text {n}'\sigma '^{-1/2} \ll 1$ the depletion interaction dominates as observed in our simulations. This explains the robustly observed $0^{\circ }$-chaining along the director. At high densities, the quadrupolar interaction becomes more relevant. We performed simulations up to very high needle densities $\rho _\text {n}'=100$ and still observe robust $0^{\circ }$-chaining as shown in Fig. 2.

## Discussion

We modeled lyotropic LC colloids as hard disks in a suspension of hard needles in a two-dimensional system. Simulations with an event-chain MC algorithm showed a chaining of disks along the director axis of the nematic needle phase. This chaining is caused by a depletion interaction due to elongated depletion zones behind and in front of the disks parallel to the director. This depletion interaction is only accessible in efficient microscopic simulations with explicit rods. Elongated depletion zones are caused by the weak planar anchoring of hard needles at hard disks. Calculating the depletion interaction with a density-dependent depletion theory and subtracting the depletion interaction we reveal a residual elastic quadrupolar interaction which is mediated by the director distortions around the disks. A quadrupolar elastic interaction is consistent with the planar anchoring. The elastic interaction is weakened because hard needles are only weakly anchored and the angular dependence is characteristically deformed because of a pronounced elastic anisotropy of the nematic needle phase.

Both depletion and quadrupolar interaction are attractive and can give rise to chaining, in principle, but the depletion favors a $0^{\circ }$-angle with the director axis while the quadrupolar interaction would favor $45^{\circ }$. In our simulations for densities up to $\rho _\text {n}' = 100$ and disk sizes up to ${\sigma ' = 10}$ we only find $0^{\circ }$-angle chaining indicating a robustly dominating depletion, which is mainly due to the weak planar anchoring. This type of chaining also governs the intermediate states in self-assembly of many colloidal disks into crystals as shown in simulation in Fig. 2.

A natural continuation of our work will be the investigation of hard spheres suspended in hard rods (such as spherocylinders) in three spatial dimensions. We believe that the two-dimensional simulation can already provide results, which qualitatively apply also to the the three-dimensional case, while giving a significant simulation speedup.

In three spatial dimensions, experiments^47, 48^ and numerical field theory^44, 46^ found quadrupolar interactions with an interaction minimum and chaining of colloidal spheres at a $30^{\circ }$-angle for planar anchoring, which corresponds to a $45^{\circ }$ in two dimensions^45^. The field-theoretic continuum approaches did not capture depletion interactions, however, while experiments in Refs.^47, 48^ were not dealing with lyotropic systems.

With respect to applications, our results show that, in lyotropic LC colloids, depletion interactions can play an important role in structure formation. By exploiting their dependence on particle shape they could provide an additional tool to control the structure formation process. Assuming that our results carry over to three-dimensional systems, we expect that the range of the depletion interaction mediated by the lyotropic LC is given by the rod length $l_0$. In order to develop similar observable consequences for chaining as in the simulations, $l_0$ should not be orders of magnitude smaller than colloidal spheres; this requires fairly long lyotropic rod-like particles. The depletion interaction dominates for weak planar anchoring as realized by hard rods and hard spheres. Experimental investigations for these types of systems are still rare at present. An ideal system to test the predictions experimentally are fd virus rods. In the experimental study in Ref.^39^ very small hard spherical particles ($\sim 20\mathrm{nm}$) in a host of hard fd virus rods (length $\sim 900\mathrm{nm}$) in the nematic phase have been studied and, indeed, an ordering parallel to the director has been found, which was attributed to depletion attraction and is in line with our observations. Our results suggest that future experiments with micron-sized colloidal particles suspended in a nematic fd virus phase could display dominant depletion interaction effects including colloidal self-assembly via parallel chaining.

## Methods

### Simulation method

#### Event-chain Monte-Carlo

To simulate hard disks in a suspension of hard needles the event-chain MC algorithm for many-body interactions is used^53, 54, 62^. This is a rejection-free MC method that performs very well for dense systems^57^, which makes it an excellent choice for needle systems in the nematic phase, for which it has been adapted in Ref.^54^. The event-chain MC is a Markov-chain MC scheme, which fulfills maximal (rejection-free) global balance rather than detailed balance. Global balance is achieved by introducing lifting moves^62^. For hard spheres or needles a lifting move is the transfer of MC displacement from one particle to another particle. This means in a MC move only one particle at a time is moved along a line until it contacts another object. For the application to hard needles, one of the two endpoints are moved and, upon collision with another needle, the remaining MC move distance is transferred to one of the endpoints of this needle. To which of the endpoints it is transferred depends on the collision point along the needle^54^. In the presence of additional disks, MC displacement is also transferred to disks if a needle collides with the disk and vice versa. Collision detection is the computational bottleneck, and we use a sophisticated neighbor list system to achieve high simulation speeds also in the nematic phase (see Supplementary Information).

#### Hard needle liquid crystal

We model the molecules of a lyotropic LC as hard needles, which consist of two endpoints connected by an infinitely thin, hard line (see Fig. 1a). For efficient sampling, the distance of the two endpoints, i.e. the length *l* of the hard needle can fluctuate around its characteristic length $l_0$ in order to allow for independent motion of both endpoints in the MC simulation; the needle length *l* is restricted by a hard potential $V_\text {n}(l)$ with $V_\text {n}(l) = 0$ for $l/l_0 \in [0.9, 1.1]$ and infinite $V_\text {n}(l)$ else, such that $l_0$ is the effective needle length. Restricting the needle length is also necessary for our neighbor lists, which accelerate the simulation.

The interaction potential $V_\text {nn}$ between two hard needles is either infinite when they overlap or zero else. This results in a lyotropic transition from isotropic to nematic upon increasing the needle density $\rho _\text {n}$. In a system with $N_\text {n}$ needles the density $\rho _\text {n} = N_\text {n}/A_\text {free}$ is defined using the available free area $A_\text {free}$ (reduced by the area occupied by disks). The simulation box is a square with edge length *L* and periodic boundary conditions. In a typical system of size $A_\text {free} = 900 l_0^2$ we simulate $N_\text {n}\sim 9000$ needles at a needle density $\rho _\text {n}'=10$.

Liquid crystalline ordering is described by the standard tensorial order parameter

$$\begin{aligned} Q_{\alpha \beta } = \frac{1}{N_\text {n}} \sum _{i=1}^{N_\text {n}} \left( \vec {\nu }^i_\alpha \vec {\nu }^i_\beta - \frac{\delta _{\alpha \beta }}{2} \right) \, , \end{aligned}$$

which is invariant under needle inversion; $\vec {\nu }^i$ is the orientation of needle *i*.

**Figure 5:**
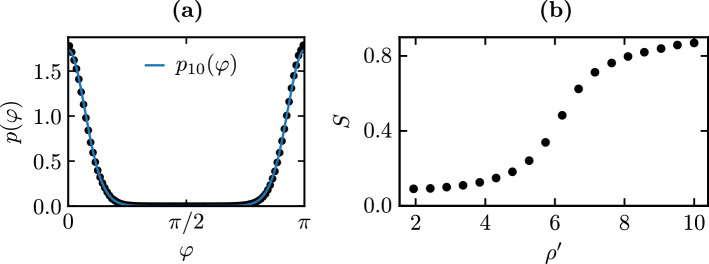
(**a**) Measurement of the probability distribution of angles $\varphi$ with the director (black dots). The probability function $p_{10}(\varphi )\sim \exp (10 \cos ^2\varphi )$ (blue line) matches the MC data for $\rho _\text {n}'=10$. (**b**) Scalar order parameter *S* as a function of the needle density ${\rho _\text {n}' = \rho l_0^2 = N_\text {n} l_0^2/A_\text {free}}$. The transition from the isotropic to the nematic phase occurs roughly at ${\rho _\text {n}' \approx 6}$ and is of Kosterlitz-Thouless type. We consider needle systems with densities ${\rho _\text {n}' \ge 10}$ as in the nematic phase and use $\rho _\text {n}' =10$ for the potential of mean force calculation and local needle density measurements. (Figure created using matplotlib 3.2.1 (https://matplotlib.org/), python 3.8.2 (https://www.python.org/), inkscape 0.92.5 (https://inkscape.org/)).

The scalar order parameter ${S \in [0, 1]}$ is calculated by diagonalizing the matrix, where ${\lambda =2S}$ is the biggest eigenvalue. The corresponding eigenvector is the director $\vec {n}$. The scalar order parameter *S* measures the degree of alignment in the system and is zero in a perfectly isotropic phase and unity in a perfectly aligned nematic phase. Figure 5 shows the order parameter *S* as a function of density for the two-dimensional hard needle system^54^; the system quasi-orders above ${\rho _\text {n}' \approx 6}$^55^. In our two-dimensional system, this transition is a Kosterlitz-Thouless transition into a quasi-ordered nematic state^56^.

### Elastic energy, weak anchoring and elastic anisotropy

For a nematic LC the state of perfect alignment is the energetically preferred configuration. But this state is almost never reached because of thermal fluctuations, boundary conditions, or topological defects. Often effective free energies from field theories are used to describe deviations from homogeneous alignment. For a director field $\vec {n}(\vec {r})$ in three dimensions, the energy of small distortions can be captured with the Frank-Oseen free energy $F_\text {FO}$^63^ with three elastic constants $K_i$, which determine the energy cost of the three different distortions splay ($K_1$), twist ($K_2$) and bend ($K_3$). In a two-dimensional system, twist distortions are not possible and, therefore, ${K_2=0}$ resulting in^60^

5 $$\begin{aligned} F^\text {2D}_\text {FO}&= \int {\mathrm{d}} A \left[ \frac{K_1}{2} (\nabla \vec {n})^2 + \frac{K_3}{2} (\nabla \times \vec {n})^2 \right] \approx \int {\mathrm{d}} A \left[ \frac{K_3}{2} (\partial _x \Phi )^2 +\frac{K_1}{2} (\partial _y \Phi )^2 \right] \end{aligned}$$

with the needle orientation field $\Phi (\vec {r})$ related to the director via $\vec {n}=(\cos \Phi , \sin \Phi )$. We assume $\Phi =0$ corresponding to $\vec {n}||\vec {e}_x$ asymptotically at the system boundary. The last approximation is the leading order in an expansion in deviations from $\Phi =0$ and neglects non-linear terms in $\Phi$^61^.

The elastic constants $K_1$ and $K_3$ can be calculated by adapting a method described by Straley^59^ to two dimensions. Strictly speaking the pronounced logarithmic orientational fluctuations in two dimensions will give rise to a renormalization at large scales^64^, which we ignore in our finite size system. We obtain ${K_1 / \rho _\text {n}'^2 k_\text {B}T = 0.086}$ and ${K_3 / \rho _\text {n}'^2 k_\text {B}T = 0.63}$ for the nematic phase with $\rho _\text {n}'=10$. This result is based on the angular distribution $p_A(\theta ) \propto \exp (A \cos ^2\theta )$ of needle orientations $\vec {\nu }=(\cos \theta , \sin \theta )$ with $A=10$ matching our MC simulations in the nematic phase with $\rho _\text {n}'=10$ (see Fig. 5). In the one-constant approximation, we assume $K=\frac{1}{2}(K_1+ K_3)$ resulting in ${K / \rho _\text {n}'^2 k_\text {B}T = 0.358}$; for this value of *K* the quadrupolar interaction (2) is shown in 4.

In order to describe colloidal disks in a nematic needle phase faithfully, we need too take into account elastic anisotropy $K_1 \ne K_3$ and a finite parallel anchoring with an anchoring strength *W*. In order to quantify the elastic anisotropy $K_1/K_3$ and the relative anchoring strength $w=W\sigma /2K_3$, we consider the needle orientation field $\Phi (\vec {r})$ around a single disk, which is suspended in a nematic phase with $\Phi =0$ asymptotically at the system boundary and quantitatively compare MC simulation data with minimization of the free energy (3) containing both the anisotropic 2D Frank-Oseen elastic energy and the anchoring potential. Free energy minimization using the linearized approximation in the Frank-Oseen part (5) results in the partial differential equation

6 $$\begin{aligned} \left( \partial _{\tilde{x}}^2 + \frac{K_1}{K_3} \partial _{\tilde{y}}^2 \right) \Phi&=0~~(\tilde{r}>1),~~~~ \partial _{\tilde{x}} \Phi + \frac{K_1}{K_3} \partial _{\tilde{y}} \Phi = \frac{w}{2} \sin \left( 2(\Phi -\Phi _0)\right) ~~(\tilde{r}=1). \end{aligned}$$

in dimensionless units $\tilde{\vec {r}} \equiv 2\vec {r}/\sigma$. Analytical solutions are only available in the one-constant approximation $K_1 = K_3$, where^65^

7 $$\begin{aligned} \Phi (r, \vartheta )&= - \arctan \left[ \frac{(\sigma /2r)^2 p(w) \sin (2\vartheta )}{1 - (\sigma /2r)^2 p(w) \cos (2\vartheta )} \right] \qquad \text {with} \qquad p(w) = \frac{2}{w}\left( \sqrt{1 + \frac{w^2}{4}} - 1 \right) \end{aligned}$$

(both $\Phi$ and $\vartheta$ are defined with respect to the asymptotic director orientation, which determines the *x*-axis). For the full problem (6) we have to resort to numerical solutions by finite element methods (using MATHEMATICA).

**Figure 6:**
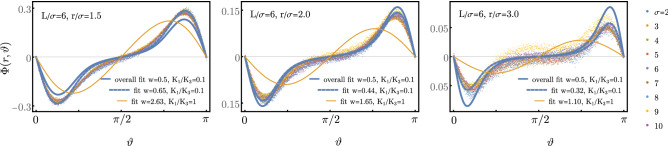
MC simulation results for $\Phi (r,\vartheta )$ as a function of $\vartheta$ and for $r/\sigma =1.5,2.0,3.0$ for parameters $\rho _\text {n}'= 10$, $\sigma =2,\ldots ,10$, and $L=6\sigma$. Data for different $\sigma$ collapse fairly well. Blue dashed lines: Least square fits of data for each $r/\sigma$ with numerical solution of the PDE (6) in the same geometry as simulation, for $K_1/K_3=0.1$, and with *w* as fit parameter. Blue solid lines: Analogous least square fit of data for all $r/\sigma =1.5-3.0$. Yellow lines: Least square fits with one-constant result (7) ($K_1/K_3=1$) with *w* as fit parameter. (Figure created using Wolfram Mathematica 12.0, inkscape 0.92.2 (https://inkscape.org/)).

In Fig. 6 we plot MC simulation data for the director orientation field $\Phi (r,\vartheta )$ as a function of $\vartheta$ (i.e., along circles) for different rescaled $r/\sigma$ and for different system sizes such that $L/\sigma =6$ remains fixed. Rescaling of lengths with $\sigma$ results in good data collapse for different $\sigma$. The characteristic shoulder of $\Phi (r,\vartheta )$ as a function of $\vartheta$ can only be explained by an elastic anisotropy $K_1/K_3\approx 0.1$. Fits with Eq. (7) from elastically isotropic one-constant approximation ($K_1=K_3$) remain unsatisfactory. We perform fits for specific $r/\sigma$ and overall fits within the whole range $r/\sigma =1.5-3.0$ with the numerical solution of the PDE (6) in the same geometry as the MC simulation (square box with $L/\sigma =6$ and director oriented along the diagonal as fixed by Dirichlet boundary conditions) using $K_1/K_3=0.1$ and *w* as fit parameter. The fit results for *w* are weakly *r*-dependent (dashed blue lines) and the result of the overall fit (solid blue line) is $w\approx 0.5$, see Eq. (4).

### Quadrupolar elastic interaction

If two colloidal disks are embedded, boundary conditions on the disk surface induce deformations in the director field and, thus, induce an elastic interaction given by the energy (5) of the deformation. There are some results on these elastic interactions in the one-constant approximation (${K = K_1 = K_3}$). For two disks with perfect planar boundary conditions there is an approximate analytical result which is the quadrupolar long-range interaction from Eq. (2)^43, 58^. Since this is a field theoretical result, the needles are assumed to be infinitely small relative to the colloids. There are no analytical results for the full two-constant free energy (5).

Therefore, we performed numerical simulations based on the finite element method (using MATHEMATICA), i.e., solving Eq. (6) for a system with two disks with distance *r* and with an angle $\vartheta$ with respect to the director axis and using the same anisotropy $K_1/K_3=0.1$ and weak anchoring strength $w=0.5$ that we determined numerically. We place both disks symmetrically in a system in a square box with $L/\sigma =10$ and with a director axis $\Phi =0$ oriented parallel to one edge of the simulation box as fixed by Dirichlet conditions at the system boundaries. The total energy of the system is the sum of elastic and anchoring energy (see Eq. (3)). The interaction energy $U_\text {quad}(r, \vartheta )$ is obtained as the difference in energy between a system containing two disks and the non-interacting system as obtained by the sum of the energies of two systems containing only one of the disks each. This results in Fig. 4b.

### Density-dependent depletion interaction

We use the results of Biben et al.^66^ and generalize them to anisotropic depletants with a rotational degree of freedom $\varphi$ to get a density-dependent depletion interaction for disks in a suspension of hard needles. We consider a system of hard disks with positions $\{ \vec {X}_I \}$ and $N_\text {n}$ hard needles with positions $\{ \vec {x}_i \}$ and orientations $\{ \varphi _i \}$. Upper case indices refer to disks, lower case indices to needles. The energy of the system is given by

$$\begin{aligned} H = \sum _{I<J} V_\text {dd}(\vec {X}_I - \vec {X}_J) + \sum _{i<j} V_\text {nn}(\vec {x}_i - \vec {x}_j, \varphi _i, \varphi _j) + \sum _{iI} V_\text {dn}(\vec {x}_i - \vec {X}_I, \varphi _i) \, . \end{aligned}$$

The disk-disk interaction is given by $V_\text {dd}$, the needle-needle interaction by $V_\text {nn}$ and the disk-needle interaction by $V_\text {dn}$. By integrating over the needle degrees of freedom one can derive the effective part of the interaction ${\mathcal V} (\{ \vec {X}_I \})$ between the disks ^66^,

$$\begin{aligned} \beta \mathcal V ( \{ \vec {X}_I \} ) = - \ln \left[ \int \prod _i {\mathrm{d}} \vec {x}_i {\mathrm{d}} \varphi _i \exp \left( - \beta \left[ \sum _{iI} V_{dn}(\vec {x}_i - \vec {X}_I, \varphi _i) + \sum _{i<j} V_{nn}(\vec {x}_i - \vec {x}_j, \varphi _i \varphi _j) \right] \right) \right] \end{aligned}$$

($\beta \equiv 1/ k_\text {B} T$). The corresponding force $\mathcal F_K ( \{ \vec {X}_I \} )$ on disk *K* is given by

$$\begin{aligned} \mathcal F_K ( \{ \vec {X}_I \} )&= - \nabla _{\vec {X}_K} \mathcal V ( \{ \vec {X}_I \} ) = - \frac{1}{N_\text {n}} \sum _l \int \rho ^{(1)}(\vec {x}_l, \varphi _l | \{ \vec {X}_I \}) \nabla _{\vec {X}_K} V_\text {dn}(\vec {x}_l - \vec {X}_K, \varphi _l) {\mathrm{d}} \vec {x}_l {\mathrm{d}} \varphi _l. \end{aligned}$$

Here, we used the single particle density of needles with angle $\varphi _l$ at $\vec {x}_l$ for fixed disk positions:

$$\begin{aligned} \rho ^{(1)}(\vec {x}_l, \varphi _l | \{ \vec {X}_I \}) = N_\text {n} \frac{ \int \prod _{i \ne l} {\mathrm{d}} \vec {x}_i {\mathrm{d}} \varphi _i \exp \left( - \beta \left[ \sum _{iI} V_\text {dn}(\vec {x}_i - \vec {X}_I, \varphi _i) + \sum _{i<j} V_\text {nn}(\vec {x}_i - \vec {x}_j, \varphi _i, \varphi _j) \right] \right) }{ \int \prod _i {\mathrm{d}} \vec {x}_i {\mathrm{d}} \varphi _i \exp \left( - \beta \left[ \sum _{iI} V_\text {dn}(\vec {x}_i - \vec {X}_I, \varphi _i) + \sum _{i<j} V_\text {nn}(\vec {x}_i - \vec {x}_j, \varphi _i, \varphi _j) \right] \right) } \, . \end{aligned}$$

By using ${\nabla _{\vec {X}_K} V_\text {dn}(\vec {x_l}-\vec {X_K}, \varphi _l) = - \nabla _{\vec {x}_l} V_\text {dn}(\vec {x_l}-\vec {X_K}, \varphi _l)}$, evaluating the sum to a factor $N_\text {n}$ and defining the average over the needle angles as ${\langle A \rangle _\varphi = \int {\mathrm{d}} \varphi A(\varphi )}$, we obtain

$$\begin{aligned} \mathcal F_{\vec {r}} ( \vec {0}, \vec {r} )&= \left\langle \int \rho ^{(1)}(\vec {r}', \varphi | \vec {0}, \vec {r}) \nabla _{\vec {r}'} V_\text {dn}(\vec {r}' - \vec {r}, \varphi ) {\mathrm{d}} \vec {r}' \right\rangle _\varphi \end{aligned}$$

for the case of two disks at $\vec {0}$ and $\vec {r}$. We use the superposition approximation ${\rho ^{(1)}(\vec {r}', \varphi | \vec {0}, \vec {r}) \approx \rho (\vec {r}', \varphi | \vec {0})\rho (\vec {r}'-\vec {r}, \varphi | \vec {0}) / \rho _\text {n}}$, where ${\rho (\vec {r}', \varphi ) \equiv \rho (\vec {r}', \varphi | \vec {0})}$ is the density distribution around a single disk. For a single disk the needles are distributed according to the direct interaction potential $V_\text {dn}(\vec {r}, \varphi )$,

$$\begin{aligned} \rho (\vec {r}, \varphi )&= \rho _\text {n} \exp \left( - \beta V_\text {dn}(\vec {r}, \varphi ) \right) . \end{aligned}$$

This finally leads to

$$\begin{aligned} \mathcal F_{\vec {r}} ( \vec {0}, \vec {r} )&\approx \nabla _{\vec {r}} \frac{1}{\rho _\text {n} \beta } \left\langle \int \rho (\vec {r}', \varphi ) \rho (\vec {r}'-\vec {r}, \varphi ) {\mathrm{d}} \vec {r}' \right\rangle _\varphi = - \nabla _{\vec {r}}U_\text {dep}(\vec {r}) \, . \end{aligned}$$

This effective potential is the density-dependent depletion interaction, which we further approximate by employing a factorization approximation for the angular averages, ${\langle \rho (\vec {r}', \varphi ) \rho (\vec {r}'-\vec {r}, \varphi ) \rangle _\varphi \approx \langle \rho (\vec {r}', \varphi ) \rangle _\varphi \langle \rho (\vec {r}'-\vec {r}, \varphi ) \rangle _\varphi }$, which is valid for the isotropic phase and the ideal nematic phase. Since we investigate the effective interaction in the nematic phase this should be a good approximation. This gives our final result

$$\begin{aligned} \beta U_\text {dep}(\vec {r})&\approx - \frac{1}{\rho _\text {n}} \int \langle \rho (\vec {r}', \varphi ) \rangle _\varphi \langle \rho (\vec {r}'-\vec {r}, \varphi ) \rangle _\varphi {\mathrm{d}} \vec {r}' = - \rho _\text {n} \int \left( 1 - \frac{\rho (\vec {r}')}{\rho _\text {n}} \right) \left( 1 - \frac{\rho (\vec {r}'-\vec {r})}{\rho _\text {n}} \right) {\mathrm{d}} \vec {r}'. \end{aligned}$$

More intermediate steps of the derivation are given in the Supplementary Information.

## Electronic supplementary material

Supplementary information

## References

[1] Russel WB, Saville DA, Schowalter WR (1990). Colloidal Dispersions.

[2] Lu PJ, Weitz DA (2013). Colloidal particles: crystals, glasses, and gels. Annu. Rev. Condens. Matter Phys..

[3] Louis AA, Allahyarov E, Löwen H, Roth R (2002). Effective forces in colloidal mixtures: from depletion attraction to accumulation repulsion. Phys. Rev. E.

[4] Asakura S, Oosawa F (1954). On interaction between two bodies immersed in a solution of macromolecules. J. Chem. Phys..

[5] Lekkerkerker HNW, Tuinier R (2011). Colloids and the Depletion Interaction.

[6] Harnau L, Dietrich S (2014). Inhomogeneous platelet and rod fluids. Soft Matter.

[7] Stark H (2001). Physics of colloidal dispersions in nematic liquid crystals. Phys. Rep..

[8] Poulin P, Raghunathan VA, Richetti P, Roux D (1994). On the dispersion of latex particles in a nematic solution. I. Experimental evidence and a simple model. J. Phys. II.

[9] Raghunathan VA, Richetti P, Roux D (1996). Dispersion of latex particles in a nematic solution. 2. Phase diagram and elastic properties. Langmuir.

[10] Terentjev EM (1995). Disclination loops, standing alone and around solid particles, in nematic liquid crystals. Phys. Rev. E.

[11] Kuksenok OV, Ruhwandl RW, Shiyanovskii SV, Terentjev EM (1996). Director structure around a colloid particle suspended in a nematic liquid crystal. Phys. Rev. E.

[12] Ruhwandl RW, Terentjev EM (1997). Monte Carlo simulation of topological defects in the nematic liquid crystal matrix around a spherical colloid particle. Phys. Rev. E.

[13] Ramaswamy S, Nityananda R, Raghunathan VA, Prost J (1996). Power-law forces between particles in a nematic. Mol. Cryst. Liq. Cryst..

[14] Ruhwandl RW, Terentjev EM (1997). Long-range forces and aggregation of colloid particles in a nematic liquid crystal. Phys. Rev. E.

[15] Poulin P, Stark H, Lubensky TC, Weitz DA (1997). Novel colloidal interactions in anisotropic fluids. Science.

[16] Ravnik M, Žumer S (2009). Landau-de Gennes modelling of nematic liquid crystal colloids. Liquid Cryst..

[17] Pergamenshchik VM, Uzunova VA (2010). Colloidal nematostatics. Condens. Matter Phys..

[18] Smalyukh II (2018). Liquid crystal colloids. Annu. Rev. Condens. Matter Phys..

[19] Muševič I, Škarabot M, Tkalec U, Ravnik M, Žumer S (2006). Two-dimensional nematic colloidal crystals self-assembled by topological defects. Science.

[20] Škarabot M (2008). Interactions of quadrupolar nematic colloids. Phys. Rev. E.

[21] Ognysta U (2008). 2D interactions and binary crystals of dipolar and quadrupolar nematic colloids. Phys. Rev. Lett..

[22] Lapointe CP, Mason TG, Smalyukh II (2009). Shape-controlled colloidal interactions in nematic liquid crystals. Science.

[23] Ognysta UM (2011). Square colloidal lattices and pair interaction in a binary system of quadrupolar nematic colloids. Phys. Rev. E.

[24] Tkalec U, Muševič I (2013). Topology of nematic liquid crystal colloids confined to two dimensions. Soft Matter.

[25] Liu Q, Senyuk B, Tasinkevych M, Smalyukh II (2013). Nematic liquid crystal boojums with handles on colloidal handlebodies. Proc. Natl. Acad. Sci. U.S.A..

[26] Pergamenshchik VM (2018). The model of elastic multipole. J. Mol. Liquid.

[27] Senyuk B, Aplinc J, Ravnik M, Smalyukh II (2019). High-order elastic multipoles as colloidal atoms. Nat. Commun..

[28] Muševič I (2013). Nematic colloids, topology and photonics. Philos. Trans. R. Soc. A.

[29] Muševič I (2017). Nematic liquid-crystal colloids. Materials.

[30] Stroobants A, Lekkerkerker HNW (1984). Liquid crystal phase transitions in a solution of rodlike and disklike particles. J. Phys. Chem..

[31] Cuetos A, Galindo A, Jackson G (2008). Thermotropic biaxial liquid crystalline phases in a mixture of attractive uniaxial rod and disk particles. Phys. Rev. Lett..

[32] Mao Y, Cates ME, Lekkerkerker HNW (1995). Depletion stabilization by semidilute rods. Phys. Rev. Lett..

[33] Yaman K, Jeppesen C, Marques CM (1998). Depletion forces between two spheres in a rod solution. Europhys. Lett..

[34] Koenderink GH (1999). Depletion-induced crystallization in colloidal rod-sphere mixtures. Langmuir.

[35] Lin K-H, Crocker JC, Zeri AC, Yodh AG (2001). Colloidal interactions in suspensions of rods. Phys. Rev. Lett..

[36] Chen Y-L, Schweizer KS (2002). Depletion interactions in suspensions of spheres and rod-polymers. J. Chem. Phys..

[37] Roth R (2003). Depletion potentials in colloidal mixtures of spheres and rods. J. Phys. Condens. Matter.

[38] Van der Schoot P (2000). Depletion interactions in lyotropic nematics. J. Chem. Phys..

[39] Adams M, Dogic Z, Keller SL, Fraden S (1998). Entropically driven microphase transitions in mixtures of colloidal rods and spheres. Nature.

[40] Lopatnikov SL, Namiot VA (1978). Interaction of macromolecules injected into a liquid crystal. J. Exp. Theo. Phys..

[41] Lubensky TC, Pettey D, Currier N, Stark H (1998). Topological defects and interactions in nematic emulsions. Phys. Rev. E.

[42] Fukuda J-I, Stark H, Yoneya M, Yokoyama H (2004). Interaction between two spherical particles in a nematic liquid crystal. Phys. Rev. E.

[43] Tasinkevych M, Silvestre NM, Patrício P, Telo da Gama MM (2002). Colloidal interactions in two-dimensional nematics. Eur. Phys. J. E.

[44] Mozaffari MR, Babadi M, Fukuda J-I, Ejtehadi MR (2011). Interaction of spherical colloidal particles in nematic media with degenerate planar anchoring. Soft Matter.

[45] Tasinkevych M, Silvestre NM, Telo da Gama MM (2012). Liquid crystal boojum-colloids. New J. Phys..

[46] Püschel-Schlotthauer S, Stieger T, Melle M, Mazza MG, Schoen M (2016). Coarse-grained treatment of the self-assembly of colloids suspended in a nematic host phase. Soft Matter.

[47] Poulin P, Weitz DA (1998). Inverted and multiple nematic emulsions. Phys. Rev. E.

[48] Smalyukh II, Lavrentovich OD, Kuzmin AN, Kachynski AV, Prasad PN (2005). Elasticity-mediated self-organization and colloidal interactions of solid spheres with tangential anchoring in a nematic liquid crystal. Phys. Rev. Lett..

[49] Schmidt M (2001). Density functional theory for colloidal rod-sphere mixtures. Phys. Rev. E.

[50] Kim EB, Guzmán O, Grollau S, Abbott NL, de Pablo JJ (2004). Interactions between spherical colloids mediated by a liquid crystal: a molecular simulation and mesoscale study. J. Chem. Phys..

[51] Rahimi M (2017). Segregation of liquid crystal mixtures in topological defects. Nat. Commun..

[52] Gârlea IC (2019). Colloidal liquid crystals confined to synthetic tactoids. Sci. Rep..

[53] Bernard EP, Krauth W, Wilson DB (2009). Event-chain Monte Carlo algorithms for hard-sphere systems. Phys. Rev. E.

[54] Harland J, Michel M, Kampmann TA, Kierfeld J (2017). Event-chain Monte Carlo algorithms for three- and many-particle interactions. EPL.

[55] Frenkel D, Eppenga R (1985). Evidence for algebraic orientational order in a two-dimensional hard-core nematic. Phys. Rev. A.

[56] Vink RLC (2014). Crossover from a Kosterlitz-Thouless phase transition to a discontinuous phase transition in two-dimensional liquid crystals. Phys. Rev. E.

[57] Kampmann TA, Boltz H-H, Kierfeld J (2015). Monte Carlo simulation of dense polymer melts using event chain algorithms. J. Chem. Phys..

[58] Dolganov PV, Dolganov VK (2006). Director configuration and self-organization of inclusions in two-dimensional smectic membranes. Phys. Rev. E.

[59] Straley JP (1973). Frank elastic constants of the hard-rod liquid crystal. Phys. Rev. A.

[60] Straley JP (1971). Liquid crystals in two dimensions. Phys. Rev. A.

[61] Galanis J, Nossal R, Losert W, Harries D (2010). Nematic order in small systems: measuring the elastic and wall-anchoring constants in vibrofluidized granular rods. Phys. Rev. Lett..

[62] Michel M, Kapfer SC, Krauth W (2014). Generalized event-chain Monte Carlo: constructing rejection-free global-balance algorithms from infinitesimal steps. J. Chem. Phys..

[63] Frank FCI (1958). Liquid crystals. On the theory of liquid crystals. Discuss. Faraday Soc..

[64] Nelson DR, Pelcovits RA (1977). Momentum-shell recursion relations, anisotropic spins, and liquid crystals in 2+$$\epsilon $$ dimensions. Phys. Rev. B.

[65] Burylov SV, Raikher YL (1994). Orientation of a solid particle embedded in a monodomain nematic liquid crystal. Phys. Rev. E.

[66] Biben T, Bladon P, Frenkel D (1996). Depletion effects in binary hard-sphere fluids. J. Phys. Condens. Matter.

