# Supplementary material for: Chaining of hard disks in nematic needles: particle-based simulation of colloidal interactions in liquid crystals
Source: Sci Rep. 2020 Jul 29;10:12718. doi: 10.1038/s41598-020-69544-4 (PMC7391704; doi:10.1038/s41598-020-69544-4)
Supplement: Supplementary file 1 — Supplementary information [file 41598_2020_69544_MOESM1_ESM.pdf]

Supplementary Information:

Chaining of hard disks in nematic needles: particle-based simulation of colloidal interactions in liquid crystals

David Müller[1], Tobias Alexander Kampmann[1], and Jan Kierfeld[1,*]

[1]TU Dortmund University, Physics Department, Dortmund, 44227, Germany
[*]jan.kierfeld@tu-dortmund.de

Effective interaction and residual elastic interaction

Fig. 1 supplements Fig. 3 from the main text and gives additional information on the effective interaction U and the residual elastic interaction $\Delta U = U - U_{\text{dep}}$ between two disks by showing cuts through the contour plot in Fig. 3 as a function of r/σ or ϑ.

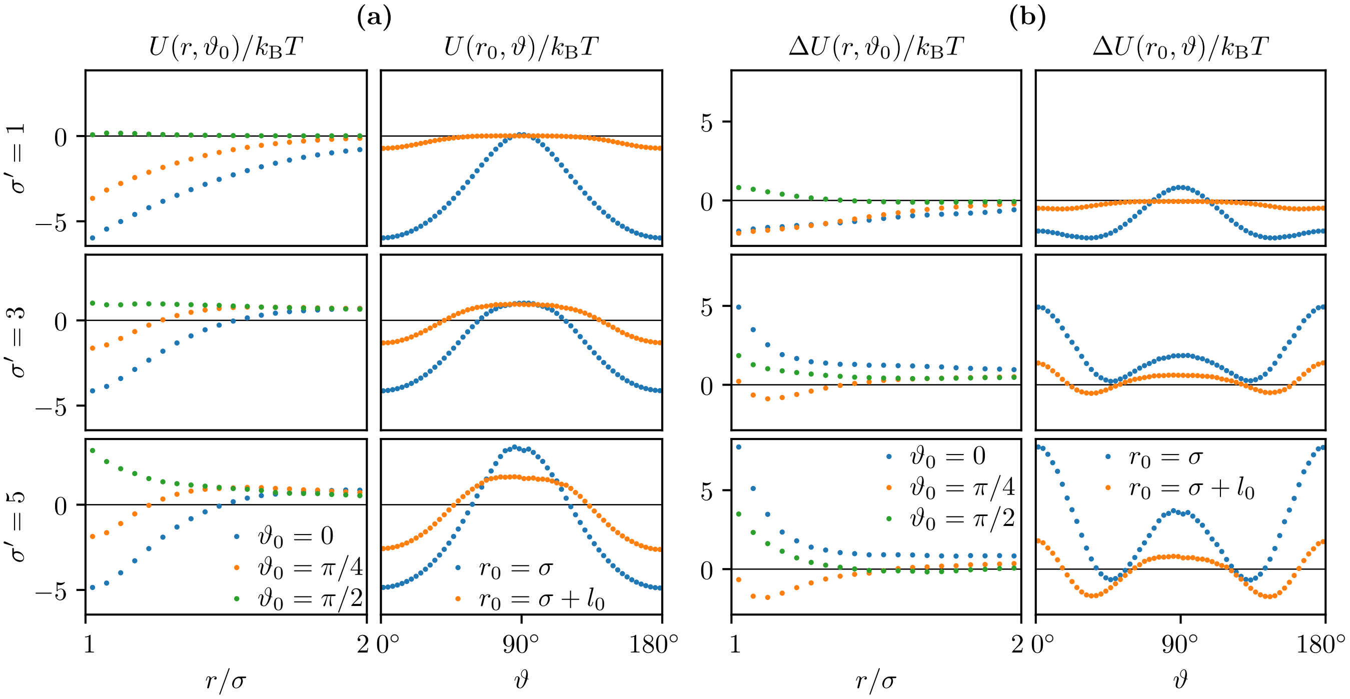

Figure 1. **(a)** Measured effective interaction of two hard disks suspended in hard needles for different disk diameters σ'. The interaction is mostly attractive with a strength around $\sim 5k_\mathrm{B}T$. The interaction minimum is at the disks' surface at $\vartheta = 0°$. The range of $U(r,\vartheta_0)$ in radial direction (left column) is of order of l_0. In angular direction (right column), $U(r_0,\vartheta)$ shows a growing repulsive area at $\vartheta = 90°$ for larger disks. **(b)** Residual elastic interaction $\Delta U(\vec{r}) = U(\vec{r}) - U_{\text{dep}}(\vec{r})$ of two disks for different diameters σ'. For larger disks the interaction shows a distorted quadrupolar pattern. For small disks ($\sigma' = 1$) the repulsive area around $\vartheta = 0°/90°$ is missing. (Figure created using matplotlib 3.2.1 (https://matplotlib.org/), python 3.8.2 (https://www.python.org/), inkscape 0.92.5 (https://inkscape.org/))

Event-chain algorithm

The event-chain algorithm is a rejection-free MC technique, which is based on global balance by introducing so-called lifting moves. For hard spheres or needles a lifting move is the transfer of a MC displacement from one particle to another particle. This means in a MC move only one particle at a time is active and moved along a line until it contacts another object. Then the remaining MC move distance is lifted to this object, which is then moved further.

Needles are represented by their two endpoints, where only one endpoint is moving at a time. In two dimensions the needle-needle interaction simplifies effectively to a collision of an endpoint with another needle. The remaining MC move distance is lifted to one of the endpoints of this needle. Therefore, we have a fluid of endpoints with an effective 3-particle interaction (two endpoints of a passive needle and the active endpoint). In Ref.[1] the generalization of the event-chain algorithm to N-particle interactions has been worked out. For needles the probability to which endpoint the MC move is lifted is proportional to the the distance to the other endpoint, i.e., it is lifted with higher probability to the closer endpoint. In the presence of additional disks, MC displacement is also lifted to disks if a needle collides with the disk and vice versa.

Effective collision detection is essential for a fast event-chain simulation. The collisions are calculated by intersections of a ray starting at the active particle (either a sphere center or a needle endpoint) with another ray, line segment, or circle. The construction and an overview of all possible cases is shown in Fig. 2 (a) and (b). To speed up the collision detection, we use a special neighbor list design. Each particle is confined to a "container", which triggers an event when the particle leaves it. Then the neighbor list is updated, which ensures that the neighbor lists are always valid. Particles are added to the neighbor lists of the other particle and vice versa when their containers overlap. This way, for different particles different container shapes can be chosen. For the needles a very narrow rectangle can be used, which limits the computational effort for calculating the

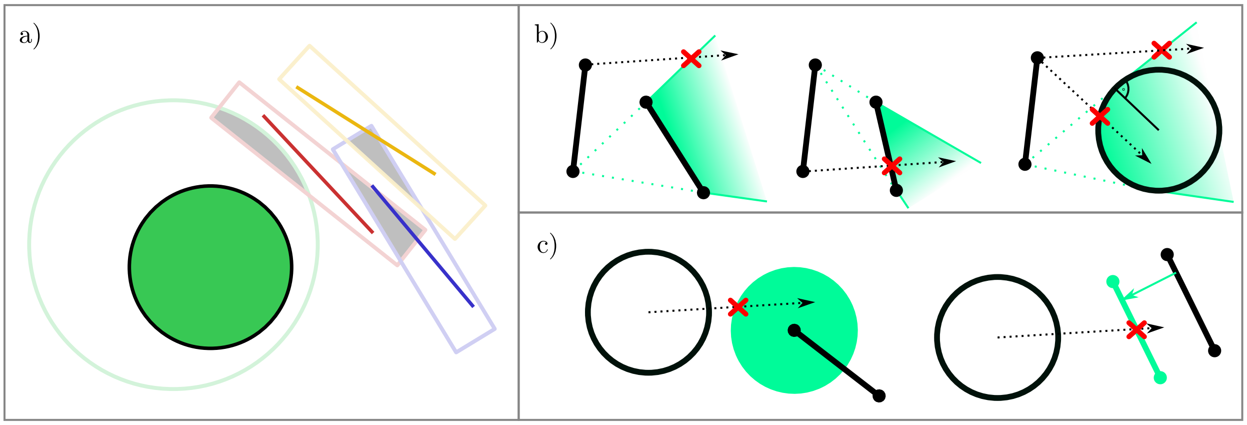

Figure 2. **(a)** Scheme for the container neighbor lists. The figure shows three needles and a sphere and their respective containers. Particle(s) are added to the neighbor lists of the other particle(s) and vice versa if their containers overlap. Arbitrary shapes address the anisotropy of needles efficiently. **(b)** Possible events in case a needle tip moves along the dashed black ray. Green lines are constructed by the inactive endpoint of the active needle and the endpoints of the hit needle or by a tangent to a hit sphere. Solid lines can trigger an event and the dashed part is to illustrate the construction. **(c)** Possible events in case a sphere moves along the dashed black ray. Left picture shows the handling of hitting an endpoint directly resembling the sphere-sphere interaction. To check for the collision with the interior of the needle, one translates the needle perpendicular to its orientation by $\sigma/2$. (Figure created iwth inkscape 0.92.5 (https://inkscape.org/))

distances to the next collision and makes the simulation significantly more efficient in the nematic phase. In particular, the anisotropy of the needles can be assigned particularly well without sacrificing any flexibility for the bookkeeping of the spheres. Even systems with a density of $\rho_\mathrm{n}' = 100$, i.e., with a mean distance of $1/100_0$, (see Fig. 2 in the main text) are possible.

To avoid numerical issues we exclude the particle (needle or sphere) which was lifted from when calculating the rejection distance. Furthermore, we optimize the updating of lists by putting them onto a collision grid.

Derivation of the density-dependent depletion interaction

Here we present the derivation of the density-dependent depletion interaction including more intermediate steps. We use the results of Biben *et al.*[2] and generalize them to anisotropic depletants with a rotational degree of freedom φ to get a density-dependent depletion interaction for disks in a suspension of hard needles. We consider a system of hard disks with positions $\{\vec{X}_I\}$ and N_n hard needles with positions $\{\vec{x}_i\}$ and orientations $\{\varphi_i\}$. Upper case indices refer to disks, s lower case indices to needles. The energy of the system is given by

$$H = \sum_{I<J} V_\mathrm{dd}(\vec{X}_I - \vec{X}_J) + \sum_{i<j} V_\mathrm{nn}(\vec{x}_i - \vec{x}_j, \varphi_i, \varphi_j) + \sum_{iI} V_\mathrm{dn}(\vec{x}_i - \vec{X}_I, \varphi_i)\,.$$

The disk-disk interaction is given by V_dd, the needle-needle interaction by V_nn and the disk-needle interaction by V_dn. By integrating over the needle degrees of freedom one can derive the effective interaction $\mathscr{V}(\{\vec{X}_I\})$ between the disks[2],

$$\beta\mathscr{V}(\{\vec{X}_I\}) = -\ln\left[\int\prod_i \mathrm{d}\vec{x}_i \mathrm{d}\varphi_i \exp\left(-\beta\left[\sum_{iI} V_{dn}(\vec{x}_i - \vec{X}_I, \varphi_i) + \sum_{i<j} V_{nn}(\vec{x}_i - \vec{x}_j, \varphi_i\varphi_j)\right]\right)\right]$$

($\beta \equiv 1/k_\mathrm{B}T$). The corresponding force $\mathscr{F}_K(\{\vec{X}_I\})$ on disk K is given by

$$\begin{aligned}
\mathscr{F}_K(\{\vec{X}_I\}) &= -\nabla_{\vec{X}_K}\mathscr{V}(\{\vec{X}_I\}) \\
&= -\sum_l \int\left[\int\prod_{i\neq l} \mathrm{d}\vec{x}_i \mathrm{d}\varphi_i \exp\left(-\beta\left[\sum_{iI} V_\mathrm{dn}(\vec{x}_i - \vec{X}_I, \varphi_i) + \sum_{i<j} V_\mathrm{nn}(\vec{x}_i - \vec{x}_j, \varphi_i, \varphi_j)\right]\right)\right] \\
&\quad\times\left[\int\prod_i \mathrm{d}\vec{x}_i \mathrm{d}\varphi_i \exp\left(-\beta\left[\sum_{iI} V_\mathrm{dn}(\vec{x}_i - \vec{X}_I, \varphi_i) + \sum_{i<j} V_\mathrm{nn}(\vec{x}_i - \vec{x}_j, \varphi_i, \varphi_j)\right]\right)\right]^{-1} \nabla_{\vec{X}_K} V_\mathrm{dn}(\vec{x}_l - \vec{X}_K, \varphi_l)\mathrm{d}\vec{x}_l \mathrm{d}\varphi_l \\
&= -\frac{1}{N_\mathrm{n}}\sum_l \int \rho^{(1)}(\vec{x}_l, \varphi_l|\{\vec{X}_I\})\nabla_{\vec{X}_K} V_\mathrm{dn}(\vec{x}_l - \vec{X}_K, \varphi_l)\mathrm{d}\vec{x}_l \mathrm{d}\varphi_l\,.
\end{aligned}$$

In the last step we used the single particle density of needles with angle φ_l at $\vec{x}_l$ for fixed disk positions:

$$\rho^{(1)}(\vec{x}_l,\varphi_l|\{\vec{X}_I\}) = N_\mathrm{n}\frac{\int\prod_{i\neq l}\mathrm{d}\vec{x}_i\mathrm{d}\varphi_i\exp\left(-\beta\left[\sum_{iI}V_\mathrm{dn}(\vec{x}_i-\vec{X}_I,\varphi_i)+\sum_{i<j}V_\mathrm{nn}(\vec{x}_i-\vec{x}_j,\varphi_i,\varphi_j)\right]\right)}{\int\prod_i\mathrm{d}\vec{x}_i\mathrm{d}\varphi_i\exp\left(-\beta\left[\sum_{iI}V_\mathrm{dn}(\vec{x}_i-\vec{X}_I,\varphi_i)+\sum_{i<j}V_\mathrm{nn}(\vec{x}_i-\vec{x}_j,\varphi_i,\varphi_j)\right]\right)}\,.$$

By using $\nabla_{\vec{X}_K}V_\mathrm{dn}(\vec{x}_l-\vec{X}_K,\varphi_l) = -\nabla_{\vec{x}_l}V_\mathrm{dn}(\vec{x}_l-\vec{X}_K,\varphi_l)$, evaluating the sum to a factor N_n and defining the average over the needle angles as $\langle A\rangle_\varphi = \int\mathrm{d}\varphi A(\varphi)$, we get

$$\begin{aligned}\mathscr{F}_K(\{\vec{X}_I\}) &= \frac{1}{N_\mathrm{n}}\sum_l\int\rho^{(1)}(\vec{x}_l,\varphi_l|\{\vec{X}_I\})\nabla_{\vec{x}_l}V_\mathrm{dn}(\vec{x}_l-\vec{X}_K,\varphi_l)\mathrm{d}\vec{x}_l\mathrm{d}\varphi_l\\ &= \left\langle\int\rho^{(1)}(\vec{r}\,',\varphi|\{\vec{X}_I\})\nabla_{\vec{r}\,'}V_\mathrm{dn}(\vec{r}\,'-\vec{X}_K,\varphi)\mathrm{d}\vec{r}\,'\right\rangle_\varphi\,.\end{aligned}$$

For the case of two disks at $\vec{0}$ and $\vec{r}$ this yields

$$\mathscr{F}_{\vec{r}}(\vec{0},\vec{r}) = \left\langle\int\rho^{(1)}(\vec{r}\,',\varphi|\vec{0},\vec{r})\nabla_{\vec{r}\,'}V_\mathrm{dn}(\vec{r}\,'-\vec{r},\varphi)\mathrm{d}\vec{r}\,'\right\rangle_\varphi \tag{1}$$

We use the superposition approximation

$$\rho^{(1)}(\vec{r}\,',\varphi|\vec{0},\vec{r}) \approx \rho(\vec{r}\,',\varphi|\vec{0})\rho(\vec{r}\,'-\vec{r},\varphi|\vec{0})/\rho_\mathrm{n},$$

where $\rho(\vec{r}\,',\varphi)\equiv\rho(\vec{r}\,',\varphi|\vec{0})$ is the density distribution around a single disk and ρ_n is the average needle density. For a single disk the needles are distributed according to the direct interaction potential $V_\mathrm{dn}(\vec{r},\varphi)$,

$$\rho(\vec{r},\varphi) = \rho_\mathrm{n}\exp\left(-\beta V_\mathrm{dn}(\vec{r},\varphi)\right)$$

resulting in

$$\nabla_{\vec{r}}\rho(\vec{r},\varphi) = -\beta\rho_\mathrm{n}\exp\left(-\beta V_\mathrm{dn}(\vec{r},\varphi)\right)\nabla_{\vec{r}}V_\mathrm{dn}(\vec{r},\varphi) = -\beta\rho(\vec{r},\varphi)\nabla_{\vec{r}}V_\mathrm{dn}(\vec{r},\varphi)\,.$$

Using this in eq. (1) we arrive at

$$\begin{aligned}\mathscr{F}_{\vec{r}}(\vec{0},\vec{r}) &\approx -\frac{1}{\rho_\mathrm{n}\beta}\left\langle\int\rho(\vec{r}\,',\varphi)\rho(\vec{r}\,'-\vec{r},\varphi)(-\beta\nabla_{\vec{r}\,'}V_\mathrm{dn}(\vec{r}\,'-\vec{r},\varphi))\mathrm{d}\vec{r}\,'\right\rangle_\varphi\\ &= \nabla_{\vec{r}}\frac{1}{\rho_\mathrm{n}\beta}\left\langle\int\rho(\vec{r}\,',\varphi)\rho(\vec{r}\,'-\vec{r},\varphi)\mathrm{d}\vec{r}\,'\right\rangle_\varphi = -\nabla_{\vec{r}}U_\mathrm{dep}(\vec{r})\,.\end{aligned}$$

This effective potential is the density-dependent depletion interaction, which we further approximate by

$$\begin{aligned}\beta U_\mathrm{dep}(\vec{r}) &\approx -\frac{1}{\rho_\mathrm{n}}\int\langle\rho(\vec{r}\,',\varphi)\rangle_\varphi\langle\rho(\vec{r}\,'-\vec{r},\varphi)\rangle_\varphi\mathrm{d}\vec{r}\,' = -\frac{1}{\rho_\mathrm{n}}\int\rho(\vec{r}\,')\rho(\vec{r}\,'-\vec{r})\mathrm{d}\vec{r}\,'\\ &= -\rho_\mathrm{n}\int\left(1-\frac{\rho(\vec{r}\,')}{\rho_\mathrm{n}}\right)\left(1-\frac{\rho(\vec{r}\,'-\vec{r})}{\rho_\mathrm{n}}\right)\mathrm{d}\vec{r}\,',\end{aligned}$$

where we used $\langle\rho(\vec{r}\,',\varphi)\rho(\vec{r}\,'-\vec{r},\varphi)\rangle_\varphi \approx \langle\rho(\vec{r}\,',\varphi)\rangle_\varphi\langle\rho(\vec{r}\,'-\vec{r},\varphi)\rangle_\varphi$, which is valid for the isotropic phase and the ideal nematic phase. Since we investigate the effective interaction in the nematic phase this should be a good approximation.

For the special case of an idealized density that is a step function and either zero or ρ_n (see Fig. 3), the effective potential essentially becomes the well-known depletion interaction $\beta U(r) = -\rho_0 A_\mathrm{ov}$, where A_ov is the overlap area of the excluded areas[3].

References

1. Harland, J., Michel, M., Kampmann, T. A. & Kierfeld, J. Event-chain Monte Carlo algorithms for three- and many-particle interactions. *EPL* **117**, 30001, DOI: 10.1209/0295-5075/117/30001 (2017).
2. Biben, T., Bladon, P. & Frenkel, D. Depletion effects in binary hard-sphere fluids. *J. Phys. Condens. Matter* **8**, 10799–10821, DOI: 10.1088/0953-8984/8/50/008 (1996).
3. Asakura, S. & Oosawa, F. On interaction between two bodies immersed in a solution of macromolecules. *J. Chem. Phys.* **22**, 1255–1256, DOI: 10.1063/1.1740347 (1954).

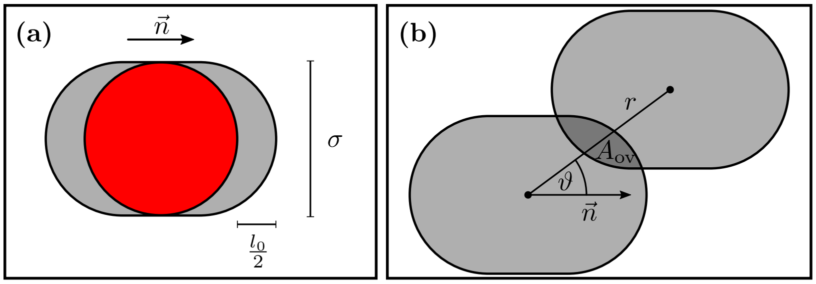

Figure 3. Idealized step-like depletion zone around a disk (a) and resulting overlap area $A_{\mathrm{ov}}(r,\vartheta)$ (b). (Figure created iwth inkscape 0.92.5 (https://inkscape.org/))
